# An exploration of healthcare providers’ experiences and perspectives of Traditional and complementary medicine usage and disclosure by Indigenous cancer patients

**DOI:** 10.1186/s12906-019-2665-7

**Published:** 2019-09-18

**Authors:** A. Gall, K. Anderson, J. Adams, V. Matthews, G. Garvey

**Affiliations:** 1 Menzies School of Health Research, Charles Darwin University, Wellbeing and Preventable Chronic Disease Division, 1/147 Wharf Street, Spring Hill, Brisbane, Queensland 4000 Australia; 2 Australian Research Centre in Complementary and Integrative Medicine (ARCCIM), University of Technology Sydney, Ultimo, Australia; 3 The University of Sydney, University Centre for Rural Health – North Coast, Lismore, Australia

**Keywords:** Aboriginal people, Cancer, Cancer care, Complementary medicine, Indigenous medicine, Traditional medicine, Communication

## Abstract

**Background:**

Traditional and complementary medicines (T&CM) are any form of medicine, practice, treatment, product, technology, knowledge system or ceremony outside of conventional medical practice that aims to prevent and/or treat illness and/or promote well-being. Alongside conventional cancer treatments, T&CM usage is increasing; with 19% of indigenous Australians with cancer reporting using T&CM. There is limited evidence surrounding T&CM use and disclosure by indigenous patients. Our aim was to explore healthcare providers’ views about usage, disclosure/non-disclosure of T&CM by Indigenous cancer patients.

**Methods:**

Semi-structured, in-depth interviews with 18 healthcare providers, including three indigenous providers, at a large urban hospital providing care to Indigenous cancer patients were conducted to explore providers’ experiences and attitudes towards T&CM use by Indigenous cancer patients. An interpretive phenomenological approach was used to thematically analyse the data.

**Results:**

Analysis revealed six themes: concern about risk; no ‘real’ benefits; perception of T&CM and conventional medicine as antithetical; barriers to disclosure; ‘patients’ choice’ a double-edged sword; and providers’ lack of knowledge about T&CM. Healthcare providers perceived discord between T&CM and conventional medicine. Most lacked knowledge of T&CM, and had concerns around negative-interactions with conventional treatments. They considered T&CM outside their role, citing this as reasoning for their lack of knowledge. Indigenous healthcare providers had greater understanding and openness towards T&CM.

**Conclusions:**

Given the potential usage of T&CM by Indigenous cancer patients, providers need a more comprehensive understanding of T&CM in order to inform discussion and facilitate effective disclosure on this topic. If indigenous Australians with cancer feel that cancer care providers are unreceptive to discussing T&CM, patient care risks being compromised; particularly given the potential for negative interactions between T&CM and conventional cancer treatments. Fostering health care interactions where indigenous patients feel comfortable to discuss T&CM usage should be a priority for all cancer care services.

## Background

Australian cancer patients in general have one of the highest survival rates in the world; however, this is not so for all groups of Australian cancer patients [1]. Cancer is the second leading cause of mortality among Aboriginal and Torres Strait Islander Australians (hereafter respectfully referred to as indigenous Australians), and most concerning is the cancer mortality gap between indigenous and non-indigenous Australians, which is increasing (1998–2015; 21% of indigenous vs 13% of non-indigenous) [1]. While reasons for this disparity are complex, there is evidence that factors such as advanced cancer stage at diagnosis [2–4], reduced access to, uptake and/or completion of treatment [3, 5–8], and higher rates of co-morbidities [3, 7] amongst indigenous patients contribute to their poorer cancer outcomes relative to non-indigenous Australians. Indigenous people tend to have a holistic concept of health, which contrasts with the biomedical model [9, 10]. This differing perspective incorporates their need for connection to culture, heritage, land and the spirits of their ancestors, which alongside the social and spiritual support that traditional healer’s provide, is seen as highly important to indigenous people [9, 10]. It is therefore likely that traditional and complementary medicine (T&CM) plays an important role in re-establishing wellness for Indigenous cancer patients, rather than solely focussing on curing the disease.

The use of T&CM alongside conventional cancer treatments such as chemotherapy and radiotherapy is increasing [11, 12]. Traditional Medicine (TM) and Complementary Medicine (CM) [14] include a broad range of practices, technologies, products, knowledge systems and approaches to preventing and/or treating illness and/or promoting well-being that are not historically associated with conventional medicine [13]. While TM refers to health care that is *indigenous* to the local culture of users (including treatments such as herbal medicines and practices provided by traditional indigenous healers), CM refers to health care, both self-administered or practitioner-led, which is often *exotic* to the culture of users (examples for indigenous Australians include massage, chiropractic and western herbal medicine) [14]. Essentially, both TM and CM are treatments provided to people outside of conventional medical practice. Whether a treatment is regarded as TM or CM is dependent on the culture and context of the user and the use [15].

A holistic approach to healthcare is fundamental to the health and wellbeing of indigenous Australians [16]. Indigenous Australians tend to view health from a holistic standpoint, which encompasses their individual spiritual, emotional and cultural needs [16–18]. This view extends beyond the physical to a whole of person approach, and beyond the individual to encompass their family, community and environment [16–18]. CM is also underpinned by a model of holism, which may be why indigenous people who lack access to TM may choose to make use of available CM alternatives. Moreover, as with all cultures, indigenous Australian culture is continually evolving and many indigenous Australians also identify other cultural groups. This makes the line between TM and CM unclear and largely superfluous. For these reasons, it was important to include CM in our discussions with providers, as their Indigenous cancer patients are potentially using CM, TM or T&CM.

There is a dearth of evidence relating to the use of T&CM among Indigenous cancer patients. In a 2015 study conducted by Adams and colleagues in Queensland, 18.7% of indigenous Australian cancer patients used at least one form of T&CM for support with their care [19]. Furthermore, there is evidence that some herbs and nutritional supplements can upregulate endogenous antioxidants that negate the effects of chemotherapy drugs [20–22]. This potential for risk has contributed to a stark divide between healthcare providers’ views on T&CM use alongside conventional cancer medicine [23–25]. Broom and Adams found oncology consultants used ‘risk’ as a means to discourage T&CM use by cancer patients, and that they held the view that the main drivers of patients to use T&CM were ‘irrationality’, ‘seeking control’ and ‘desperation’ [25]. These findings highlight potential barriers for cancer patients to disclose their use of T&CM with their healthcare providers [25].

The importance of open and effective patient-clinician communication is paramount in modulating the potential risks and benefits associated with the use of T&CM in the cancer setting [25, 26]. The communication gap between healthcare providers and indigenous Australians has a significant impact on health outcomes [27–30]. Cass and colleagues used qualitative methods to observe both healthcare providers and indigenous renal patient communicative interactions, along with face-to-face interviews, to explore factors that may lead to ineffective communication in clinical settings [27]. They found that there was rarely a shared understanding of the key concepts involved in the patients’ healthcare, and that this miscommunication often went unnoticed. Similar findings were reported in a study by Lowell and colleagues where interviews with indigenous chronic disease patients revealed that patients felt they were not receiving the detailed information about their healthcare they needed and that the information was willingly being withheld from them [28]. These findings highlight how pervasive clinical miscommunication can be with indigenous patients and non-indigenous providers, which undermines trust in the patient-clinician relationship which is vital to the success of healthcare [28].

A handful of studies have identified factors that hinder patient-clinician communication with indigenous Australian cancer patients. These factors include healthcare providers self-reporting that they struggled to communicate with their Indigenous cancer patients, especially when English was the patients’ second or third language [26]. This is concerning given that healthcare providers in the cancer-care setting often convey complex information to their patients that is imperative to their care [26]. Meiklejohn and colleagues found that healthcare providers reported difficulties in understanding Indigenous cancer patients’ worldviews and the complexity behind their connection to land and family [26]. This finding corresponds with Shahid and colleagues that Indigenous cancer patients felt healthcare providers ignored the importance of their connections to land and family, which contributed to the breakdown of trust in the clinical relationship [31]. Other factors that affect patient-clinician communication include the constrains around appointment times, with lack of time being seen by the healthcare providers as a significant barrier to effective communication [26].

Understanding healthcare providers’ knowledge and understanding of T&CM use among their Indigenous cancer patients, and their openness to patient disclosure of T&CM use, is an important first step in building an evidence base in this area [31]. The aim of this study is to explore healthcare providers’ experiences and perspectives relating to usage, disclosure and non-disclosure of T&CM by Indigenous cancer patients. Indeed, exploring the cancer-care setting that Indigenous cancer patients experience will provide insight into the possible barriers and facilitators they face when making decisions around disclosure and non-disclosure of T&CM use with healthcare providers.

## Methods

### Participants and data collection

We employed purposive sampling to ensure we captured views on use and disclosure of T&CM from a broad range of healthcare roles at a large urban hospital in Queensland. Inclusion criteria was any healthcare providers (e.g. social workers, nurses, oncologists) involved in the care of Aboriginal and Torres Strait Islander cancer patients. Once identified, the providers were initially informed about the study by a clinical nurse consultant, who then informed the study group of any providers who had shown an interest in being involved in the study. Upon contacting the providers again, those who indicated a willingness to participate in the study were then contacted by an experienced study interviewer to organise a time to meet. The interviewer confirmed in person with the provider their willingness again to participate, gained their formal consent and then conducted the interview. Data collection was undertaken from November 2016 to May 2017.

### Study design

The study employed qualitative research methods and the interviews were guided by a semi-structured interview schedule. The interview schedule was initially assessed and amended for flow, wording and ease of use in mock interviews with non-providers. It was then pilot tested with one provider for clarity of content and appropriate terminology. No changes were made to the schedule following this pilot interview, and the data from this interview was included in the current analysis.

The interview schedule also included a definition of T&CM (*Complementary and Alternative medicines (or CM) refer to any therapies, medicines, herbal and nutritional supplements that sit outside of the dominant health system. For example, meditation, Homeopathy, Naturopathy, Traditional Chinese Medicine. Traditional Aboriginal Medicines include the use of singing/chanting, bush medicine, traditional healers and external remedies prepared for healing or prevention purposes*) and a broad outline of topics to be discussed, but also allowed the providers ample opportunity to both describe and explain their experiences and perceptions in their own words and on their own terms as well as introduce new topics for discussion.

Interviews were undertaken face-to-face and all interviews were audio recorded with the providers consent. Field notes were taken where relevant. Basic demographic information was also collected from the providers relating to their ethnicity, sex, job role, and length of time they had worked in the role.

### Data analysis

All interviews were transcribed verbatim, de-identified and checked for accuracy and imported into NVivo11 [32] for analysis. As data coder (AG) has previous knowledge of T&CM, we introduced a second data coder (KA) with no prior knowledge of this area, by doing this and by undertaking the following process, we were able to reduce expert bias and therefore strengthen the validity of our analysis. Each transcript was independently read by two data coders (AG and KA). Major themes were identified through an iterative process and examples were documented separately by each coder. Concept maps were used to compare and contrast categories throughout the research process and to consolidate these categories into themes that related to the research aims. The coders (AG and KA) collaboratively negotiated themes into a common final set to be analysed. Using these themes the coders (AG and KA) made quote selections, ensuring the most illustrative quotes covering all provider views were used. The interview content in each of these themes was analysed using an interpretive phenomenological analysis approach [33], which offers insights into the “lived experience” of the participants and an understanding of how people make sense of a phenomenon within a given context. Identified themes and quote selections were verified by a third independent coder (GG).

### Ethical considerations

Ethical approval was obtained from the Northern Territory Department of Health and Menzies School of Health Research Human Research Ethics Committee (HREC2015–2413) and the Mater Hospital Brisbane Human Research Ethics Committee (HREC15MHS55). All procedures were in accordance with the ethical standards of the institutional and/or national research committee and with the 1964 Helsinki declaration and its later amendments or comparable ethical standards. Informed consent was obtained from all individual providers included in the study. No quotes were labelled to ensure the anonymity of all providers included in the study was upheld.

## Results

A total of 18 interviews were undertaken with a range of healthcare providers including: senior registrar (*n* = 1), medical consultant (*n* = 1), nurses (*n* = 4), pharmacists (*n* = 3), Aboriginal liaison officers (*n* = 2), senior social worker (*n* = 1), physiotherapists (*n* = 2), pastoral care workers (*n* = 3) and a clinical nurse consultant (*n* = 1). Of those interviewed, 15 were female (83%), three were indigenous Australian (17%), and most (*n* = 12) had been employed 4+ years in their current role.

Six key themes were identified relating to the study aims: concern about risks using T&CM; no ‘real’ benefits of T&CM; perception of T&CM and conventional medicine as antithetical; barriers to disclosure; ‘patients’ choice’ is a double-edged sword, and; providers’ lack of knowledge of Traditional indigenous Medicine.

### Concern about risks associated with using T&CM

A common thread throughout the interviews was a concern about potential risks of using T&CM in conjunction with conventional cancer treatments. Specifically, health care providers mentioned interaction effects between drug-herb and drug-nutrient substances, as well as compounding effects of using nutrients or herbs that perform the same action as a drug. One provider illustrates this common concern around interactions, after explaining that her cancer patients mainly use vitamins and herbs *“Usually when I admit a patient I ask “Did you bring any tablets with you? or over the counter … prescription?” because we have to know [in case of] some sort of interaction with medication, even [if the tablets] are natural but still [not] safe to give it [with the] medication prescribed.”*

One compounding effect that was mentioned by several providers was of fish oil thinning the blood. Several providers stated that they recommend patients stop using fish oil before surgery, as when compounded with the use of drug-based blood thinners, the risk of bleeding increases. However, one provider explained that she assumes that the patient’s general practitioner will already have discussed this with the patient before coming to hospital, so she does not usually raise it.

*“So even things like fish oil, if you take fish oil we say, stop that seven days before you have an operation, because it can thin the blood.”*

*“I would be concerned especially most of our surgical patients they are on blood thinners post-surgery and things like that. I would be concerned if that would interfere … it would further thin the blood as well and increases their risk of bleeding.”*

There was also a reported lack of evidence available to providers about the nature, efficacy, risks and benefits of T&CM. Several providers explained that this makes it difficult to determine what position to take on the use of T&CM: “*… it’s very hard because there is not a lot of literature in terms of drug interactions which is … my main concern …*. *It depends on what the complementary medicine was and if there was evidence to support stopping or starting or continuing that would help to guide my decision …*” *.* Another provider reiterates this reliance on evidence-based medicine by stating: “*… We just stick to evidence-based medicine.”*

This focus on risk overshadows the potential benefits of T&CM, illustrated by the comment: “*… the truth of the matter is we probably have no interest, that’s awful, but the medical structure has no interest in anything that isn’t either a form of ingestion or topically that could affect you or interact … we’re only concerned with the things that will influence what we’re doing for the patient, not exactly holistic but that’s it.”*

### No ‘real’ benefits associated with T&CM

When asking providers about benefits to patients using T&CM, responses were mixed, with some giving a detailed response and others simply stating they did not know of any benefits. Of those that gave detail of the benefits of T&CM, an incongruity in their views was evident. While several providers spoke of benefits to mood, emotions, self-esteem and sense of control, these positives were couched in language suggestive that these ‘benefits’ were merely placebo effects and had no ‘real’ effects on the cancer itself.

*“I’ve got vague ideas that the actual traditional cultural meanings, people get benefits from that just because they’re engaging in their culture. I’ve heard of this before but I don’t know any physiological reasons other than – more along the – not placebo, but along those lines that can be helpful to people because they feel like they’re taking ownership, doing something for themselves, and it’s more along those kind of lines. But that’s all I know; this vague idea of, it helps them because they’re doing something for themselves.”*

While another provider immediately responds to the question of benefit *“No”* followed directly after with *“Only emotionally, or spiritually, she’s complete”* illustrating the common distinction between ‘real’ benefits in fighting the cancer and the lesser valued benefits to emotional wellbeing. As one provider reports, *“I think the best [benefit] would be a sense of control in decision making and being responsible for their own health and trusting what they know.”*

This distinction between ‘real’ benefits of conventional medicine and ‘placebo’ benefits of T&CM was underpinned by the perceived lack of evidence and providers’ lack of knowledge about T&CM, which greatly influence providers’ opinions of T&CM.

### Common perception of T&CM and conventional medicine as antithetical

Another common theme embedded in the language of the providers was that T&CM and conventional medicine are discrete, antithetical concepts. One provider alluded to this, firstly referring to anything the patient takes that is not conventional medicine as being *“unusual”* then elaborating *“I’m not really a fan of mixing herbal with our regular medication.”* The language of *unusual* versus *regular* is suggestive of a right versus wrong conception of these paradigms. Another provider gave a positive interpretation of this difference: *“I think it’s just a mindfulness of people treating them. That they do have different traditions and beliefs. And I think that they should be also explored and accepted from the team treating them.”*

### Barriers to disclosure

Providers were asked about talking with patients about T&CM and whether they found patients to be open to discussing T&CM. Providers who thought patients were open to discussing T&CM, described that *rapport* must be established to achieve a willingness to disclose. One provider responded: *“[patients are open about T&CM use] if asked the right way. And I tend to – specifically if I am with an indigenous patient and I am doing a medication history interview, I like to establish rapport first …*” *.* In contrast, among those providers who did not discuss T&CM with patients, there was a common sense of not wanting to know. A few providers stated that it is *“not my role”*, with one nurse referring patients to other providers if the patient spontaneously disclosed T&CM use: *“If they ever mention to me [T&CM use] I will always actually ask them to consult with their treating team …*” . One provider spoke about patients’ reluctance to disclose their T&CM use for fear of disapproval, which is a possible barrier to disclosure: “*… they see a hospital as very western medicine and therefore not open to other views and often people will not – they don’t tell the doctor they’re even on medications or they’re on minerals, vitamins, whatever, they won’t tell them because they think that we would disapprove and that happens”.* When considering TM specifically, two providers alluded to a concerning issue where the lack of provider knowledge of TM, coupled with the likelihood that patients would not disclose anything they see as irrelevant to conventional treatment, likely results in self-perpetuating non-disclosure of patients and unawareness of providers: “*… they’re always asked but whether they would actually consider what they’re, say, singing and chanting, would they consider that as part of what we’re expecting …*” *.*

### ‘Patients’ choice’ is a double-edged sword

The idea that it is the patients’ choice whether they choose to use T&CM is a complex and value-laden issue, which was illustrated throughout the provider interviews. The concept of choice presented itself as a double-edged sword; on one hand providers spoke of the positive aspects of T&CM use being empowering for the patient, while on the other hand providers viewed that by engaging in non-conventional medicines, the patient was exposing themselves to risks outside of the control of the mainstream providers. The inability to control these risks for patients was clearly a concern for providers, however, the rights of the patient to make their own determination was acknowledged by several providers.

*“We explain our medical point of view, if they want to go off and try alternative medicines we accept that that’s their choice and they have control over their health and if they turn to – want to return, if that medicine has failed and they want to come back we – they are always welcome and we treat them then if we can.”*

*“It’d be like me saying you can’t have chemo medication, it’s not my place, it’s the patients. If that patient had made that – had gone to her family, and they’ve gone to do that [engaged in T&CM use], that’s the family business.”*

One provider had a very strong response throughout her interview in regard to cannabis/marijuana use for symptom relief as being a patient’s choice, stating several times throughout the interview that *“I have no judgement or opinion on it.”* Another provider echoes this view when they stated: “*… we have a lot of cancer patients [that] tend to seek the alternative medicine, a lot of them with the cannabis oil at the moment, that’s a big, big way that a lot of people are going; if that’s their choice, that’s their choice.”*

Further complicating this concept of choice is the perceived chasm between T&CM and conventional medicine that is apparent in all the interviews with providers, with one provider stating they know what is ‘best’ for the patient, and putting the onus on the patient to make the right choice in the eyes of the provider: *“we suggest what’s best, most appropriate, depends on patient acceptance, I suppose, what they would take as the recommendation”.* This assumption that conventional medicine is the ‘best’ choice and that the patient is expected to ‘accept’ this is the underlying premise of evidence-based medicine that underpins the reported views in all of the interviews with providers. When this assumption is coupled with the perceived ‘otherness’ of T&CM, it is easy to see how the use of T&CM comes to be regarded by providers as risky and potentially hazardous.

### Providers’ lack of knowledge and understanding of Traditional indigenous medicine

All providers’ self-reported their knowledge and understanding about CM was varied, and the non-indigenous providers reported they had very little to no knowledge of or understanding of TM.

*“I know nothing about the Aboriginal traditional medicines”*

*“I even don’t know what they are”*

*“The only thing I’ve seen here is people burn different oils down here – because we have burners in all of our rooms. That’s it, I don’t know if that’s a tradition they they’re doing, or is it just ‘cause we have a burner and they go, “Oh, that would be nice to do that.” So, I don’t know anything in particular.”*

One of the three indigenous providers, that all self-reported having knowledge about TM, elaborated on the lack of widespread understanding of TM among other providers: *“I’d suspect that my colleagues would not know a lot about bush medicines or the fact of singing and smoking ceremonies and things like that, I don’t think they would appreciate that that is part of a healing treatment option that indigenous patients might use …*”.

The fact that providers reported generally knowing less about TM than CM, suggests that they would be unlikely to recognise or be aware if indigenous patients’ were using TM as part of their cancer-care. One provider alluded to the reasons for the lack of knowledge around TM when she stated: “*… my knowledge is lacking … it’s too time consuming to go and investigate traditional medicines as well, you know...*” indicating they only have time to concentrate on conventional medicine as part of their role.

## Discussion

To our knowledge, no previous research has explored health provider’s experiences of and attitudes towards indigenous Australian cancer patients’ T&CM usage and disclosure. Our findings suggest there is a commonly held perception that T&CM and conventional medicine are oppositional rather than complementary in nature, which is reflected in the knowledge, understanding and perspectives about T&CM of the healthcare providers. Most providers in this study conceded they have little knowledge about T&CM, with their main concerns relating to the potential negative interactions when used concurrently with conventional cancer treatments. There was an evident disconnect between the evidence-based approach of the providers interviewed, and the holistic benefits that T&CM can confer to patients. Some providers reported the view there are no ‘real’ benefits of T&CM use, likening its effects to a placebo. However, this overlooks some important non-physiological benefits that T&CM provides, such as patient involvement in decision making and the benefits of practicing and engaging in culture, which have been shown as beneficial in building the self-efficacy of patients [18].

Providers cite the lack of evidence around T&CM as reasoning for their lack of knowledge and wariness surrounding the use of T&CM. Research suggesting possible usage and self-reported benefits of T&CM in cancer is emerging [34, 35] however, strong evidence remains limited. Moreover, some providers stated they do not have the time to seek out the evidence and/or feel that T&CM is not part of their role, which further widens the separation of T&CM and conventional medicine. Interestingly, indigenous providers, while still concerned about potential risks, revealed a greater depth of understanding and knowledge of T&CM, especially concerning TM. This may in part be owing to their cultural and personal understanding of the differences in health paradigms adhered to by indigenous people and that of conventional medicine [36, 37]. This greater depth of understanding was reflected in their responses around communication, explaining how they use rapport building in the first instance to facilitate open responses from their patients. Similarly, non-indigenous providers who also cited rapport building as an important first step to communication also felt their patients were more open about their T&CM use. These results infer that open communication may be achieved through building rapport and providing a safe environment for the patient, which may in turn increase the disclosure of T&CM.

Our findings support the findings of previous studies involving non-Indigenous cancer patients that concerns around potential risks contributes to the divide between providers who support T&CM use and integration with conventional medicine, and those who do not [23–25]. Broom and Adams found that oncology consultants used their perceived risk of CM as a means to discourage its use by their patients, although the consultants indicated they had little knowledge of CM or the risks [25]. Furthermore, oncology nurses saw themselves as CM advocates, although this was discouraged by the oncology consultants. Due to their lack of knowledge of risks associated with CM use, they found the providers’ individual beliefs about CM led to their decision to discourage its use. Indeed, Shorofi and colleagues found a positive association between nurses’ knowledge and their attitudes towards CM, further showing this link between individual’s beliefs about CM and their attitude towards it [38]. The importance of open and effective patient-clinician communication is paramount in modulating the potential risks and benefits associated with the use of T&CM in the cancer setting [25, 26]. Previous studies have identified several factors that hinder patient-clinician communication in regards to indigenous patients [26, 31]. For example, in a 2016 study conducted by Meiklejohn et al., they found that the main barriers to cancer care for indigenous people were related to challenges with communication, the health system and coordination of care, issues around individual and community priorities and views of cancer treatment and health professional judgement [26]. However, while these different factors may be present in the current setting, our findings suggest that the large divide in health paradigms and general lack of provider’s understanding and knowledge of T&CM, contribute to communicative discord in the present group.

As with any study, this study has some strengths and limitations. The inclusion of a range of providers involved in cancer-care for indigenous patients from a large urban hospital strengthens these findings; however our limitations are that these results may not be representative of all providers, both in and out of the cancer-care setting and these findings may not be representative of providers in rural and remote locations.

## Conclusions

Given the possible usage of T&CM by Indigenous cancer patients, our findings suggest that the limited understanding of healthcare providers of the risks, benefits and usage of T&CM among their patients, should be addressed in order to inform discussion and disclosure around this topic. Furthermore, providers need to be mindful of creating a safe space for disclosure by helping the patient feel comfortable when asking the question about T&CM use. Our findings suggest this may be achieved through initial and ongoing culturally-competent rapport building. In addition to building a stronger evidence base on the efficacy and risks associated with T&CM use in cancer-care, clinical practice would benefit through research investigating indigenous patients’ experiences and perspectives of as well motivations for using T&CM.

## Data Availability

The datasets generated and analysed during the current study are not publicly available due to the easy identification of included participants, and no ethical approval has been given to share the full datasets outside the research group listed on the original ethics application. However, the corresponding author can be contacted to further explore the data upon request.

## Abbreviations

CM: Complementary medicine
T&CM: Traditional and complementary medicine
TM: Traditional medicine
